# Increasing Neurosurgical Resident Research Productivity Through Cultural Shift: Choosing Carrots Over Sticks

**DOI:** 10.7759/cureus.69278

**Published:** 2024-09-12

**Authors:** Mustafa Motiwala, Erin Miller, Michael Herr, Ahmed Motiwala, Amro Amro, Vincent Nguyen, Andrew J Gienapp, Paul Klimo, L. Madison Michael

**Affiliations:** 1 Neurosurgery, The University of Tennessee Health Science Center, Memphis, USA; 2 Neurosurgery, Atrium Health Carolinas Medical Center, Charlotte, USA; 3 Cell, Developmental and Integrative Biology, Heersink School of Medicine, University of Alabama, Birmingham, USA; 4 Public Health, Brown School at Washington University, St. Louis, USA; 5 Podiatric Medicine, New York College of Podiatric Medicine, New York City, USA; 6 Neurosurgery, Keck School of Medicine, University of Southern California, Los Angeles, USA; 7 Neuroscience Institute, Le Bonheur Children's Hospital, Memphis, USA; 8 Neurosurgery, Semmes Murphey Clinic, Memphis, USA

**Keywords:** authorship, bibliometric, citation, h-index, publication, research, resident

## Abstract

Introduction

As part of the Milestones Initiative of the Accreditation Council for Graduate Medical Education (ACGME), residents in neurosurgery are expected to participate in either clinical research or basic science research. Therefore, each neurosurgical training program must offer the support and opportunity to achieve this goal. In 2012, a structured effort to promote a resident culture of research was introduced into the authors’ neurosurgery residency training curriculum. This study reviews this experience over the last decade.

Methods

Data were collected from the authors’ departmental neurosurgery website and Scopus to create a database of neurosurgical residents who graduated 10 years before and after 2012 and their publication output. Bibliometric measures were collected for all articles published by residents. Results were compared between residents who graduated before and after the introduction of the research initiative.

Results

A total of 127 publications were analyzed from 37 residents, constituting 174 authorships. There was a statistically significant increase in the number of publications per resident (P < 0.001), citation number per author (P = 0.002), and author h-index (P < 0.001) after implementing the initiative. There were no significant differences in the pre-residency and baseline demographic variables between the two groups.

Conclusion

This study relates the experience of initiating a research culture at the authors’ neurosurgery training program, which did not emphasize scholarly productivity historically. The effort focused on creating a culture of curiosity as opposed to formal requirements. The results provided evidence that this strategy yielded a significant increase in academic output and impact. These findings have important implications for neurosurgical training programs.

## Introduction

The Accreditation Council for Graduate Medical Education (ACGME) mandates that residents meet the challenges of an ever-changing field of medicine across many competencies, such as clinical knowledge, patient care, and system-based practice [1]. However, a survey of surgical program directors and trainees revealed that resident achievement in scholarly activity, which is a provision of the ACGME’s Common Program Requirements (IV.D.2) [2], ranked much lower than mastery of other core competencies [3]. With this emphasis on scholarly endeavors, programs must adapt to support trainees in developing necessary research skills.

In the ACGME Milestones initiative, residents in neurosurgery are expected to participate actively in research-clinical research or basic science. A dedicated milestone that describes benchmarks for resident research is found under the Practice-Based Learning and Improvement competency. In a specialty known for high academic achievement, internal and external research expectations for neurosurgery residents reflect the competitiveness of the field. Data presented by the National Resident Match Program from 2020 ranked neurological surgery applicants as the highest producers of scholarly activity based on the mean number of abstracts, presentations, and publications among matching allopathic medical school seniors [4]. Indeed, research productivity is a lauded attribute in applicants to neurosurgery residency and fellowship positions as it reflects dedication and work ethic, professional engagement, and an ability to contribute meaningfully to the academic culture of the field [5]. Moreover, research is not simply a prerequisite for a successful match into neurosurgery, but also a continued expectation throughout training as scholarly work during residency has been identified as highly predictive of future academic advancement [6,7].

As a cornerstone of today’s neurosurgical training, academic involvement is growing across residency programs with some institutions adopting research requirements guided by the field’s accrediting and certifying bodies [1,8]. Certain neurosurgery programs incorporate structured and protected research time in training while others still allow residents to determine their own level of involvement with academic pursuits. Numerous general surgery residency training programs have reported increased productivity after implementing organized research initiatives [9-13], but there is a paucity of data reporting similar metrics in neurosurgery. Specifically, no outcomes from such initiatives have previously been reported on within the neurosurgical literature.

In 2012, a structured effort to promote resident research was introduced into the training curriculum of our institution’s neurosurgery residency program. Now eight years on, the availability of publication data for the generation of residents who underwent this transition offers the opportunity to reflect on these changes. The present study aims to review our program’s “cultural shift” from implicit expectations to explicit directives and the associated impact on resident scholarly productivity. Using established bibliometric measures [7,8,14-16], we sought to quantify the magnitude of this change, which we hope can serve as a blueprint for other programs working to foster their own research culture.

## Materials and methods

Structured intervention

During the fall of 2012, the Department of Neurosurgery at the University of Tennessee Health Science Center, Memphis, instituted a formalized effort to achieve academic productivity among its residents. On the infrastructure side, the program introduced a faculty member as a dedicated Research Program Director, a Research Coordinator familiar with institutional review board processes, and a Medical Editor with American Medical Writers Association certification in medical editing/writing and substantial prior publishing experience. The Research Program Director was in charge of organizing a schedule of quarterly research symposia as part of the residency program’s regular academic conferences so that all residents could present new scholarly projects and updates on existing projects at regular intervals throughout the year. All neurosurgical faculty were encouraged to attend the meetings. Residents were required to attend and participate in these meetings; however, specific publication targets were not defined. Residents were also required to self-direct their projects and ask for faculty mentoring and/or assistance when needed. No additional “dedicated” or “protected” time or funding was allotted to residents for research but travel and lodging for academic purposes remained supported through the university.

Data collection

During March-April 2023, data were obtained from our university’s Neurosurgery Department website and Scopus (Elsevier, Amsterdam, Netherlands) to create a database of residents who graduated 10 years before and 10 years after the research program was introduced. Previous work by our group has indicated that Scopus is more accurate in individual bibliometric calculations when compared to competitor databases [14]. Demographic information was collected, and bibliometric measures for each resident were recorded.

Primary outcome measures included authorship of peer-reviewed publications per resident during their training. Book chapters were not included. Each article was further evaluated to confirm peer review, categorize the article as either basic science or clinical research, define the subject of the article, and identify the publishing journal. Article subjects included tumor, vascular, spine, trauma, functional, pediatric, skull base, or other. The other category included papers on general surgical approaches, neurocritical care, infection, imaging, or bibliometrics. Secondary outcome measures included residency h-index, citation number per author, and journal impact factor of each publication. The h-index is defined as an individual having h papers with at least h citations [17] and was calculated using only articles published during residency. This metric is especially useful in academic medicine as it links the quantity of publications with impact [14]. The journal impact factor was determined by searching the Web of Science Group’s Master Journal List for 2021-2022 [18].

Data analysis

All collected data was exported into Microsoft Excel (version 16.56, Microsoft Corporation, Seattle, Washington) for organization and then into GraphPad Prism (version 9.3.1, GraphPad Software, San Diego, CA) for analysis. Tests for normality were performed using the Shapiro-Wilk test. Nonparametric tests included the chi-square and Mann-Whitney U tests to evaluate data with nominal and continuous variables, respectively. Significant values were defined as P < 0.05.

## Results

Resident characteristics

Pre-residency and demographic factors were examined for residents training before and after the start of the research program and are shown in Table 1. There were 19 residents before the initiation of the research program and 18 residents after. The median number of residents was two per year (range, 1-3). Analyzed factors included gender, medical school, PhD status, and number of publications prior to residency. There were no significant differences in terms of these variables across each of the groups.

**Table 1:** Resident demographic (N = 30) and authorship characteristics before and after implementation of residency research program.

Characteristic	Before implementation	After implementation	P-value
Male gender, n (%)	19 (100.00)	18 (100.00)	1.000
Home medical students, n (%)	6 (31.58)	5 (27.78)	0.852
PhD, n (%)	1 (5.26)	1 (5.56)	0.970
Pre-residency publications, mean (SD), range	0.79 (1.44), 0–5	0.94 (1.47), 0–5	0.341
Authorship positions, n (%)			
First	9 (47.36)	62 (40.00)	0.695
Second	3 (15.79)	38 (24.52)	0.494
Middle	5 (26.32)	55 (35.48)	0.569

Resident authorship

**Figure 1:**
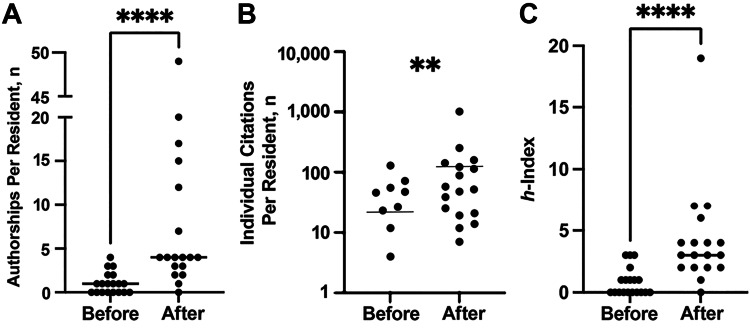
Before and after comparisons of authorships per resident, citations per resident, and h-index. (A) Authorships per resident before and after implementation of the residency research program. (B) Individual citations per resident before and after implementation of the residency research program on a log scale. (C) Individual resident h-index before and after implementation of the residency research program.

We collected 127 unique publications from 37 residents accounting for a total of 174 authorships. The 19 residents prior to the research program contributed 18 unique publications (19 authorships, mean=1.05, SD=1.22, 0-4), and the 18 residents after contributed 109 unique publications (155 authorships, mean=8.61, SD=11.62, 0-49), which constituted a statistically significant increase in the number of authorships per resident (P < 0.001) (Figure 1A). There was a statistically significant increase seen in the number of citations per resident with an average of 21.79 (SD=34.59, 0-129) citations before and 120.83 (SD=230.84, 0-1006) citations after (P = 0.002) (Figure 1B) and each resident h-index (calculated with only publications produced during residency, as described above) which averaged 0.84 (SD=1.12, 0-3) prior to and 4.22 (SD=4.14, 0-19) post introduction of the research program (P < 0.001) (Figure 1C). The authorship position (first, second, or other) for each paper published by a resident was assessed, and no significant difference was found between the proportion of first, second, or middle (i.e., not first, second, or last) authorship ranks from before and after the implementation of the research program (Table 1).

Article characteristics

Table 2 compares the type and subject of articles written by residents before and after the introduction of the research initiative. Overall, clinical research was more common than basic science, as it comprised 78% of papers before and 96% of papers after the research requirement. Pediatric neurosurgery was the most common subject of resident publications with 33% of all papers before and 31% of all papers after the start of the research program. Article subjects varied and a wider distribution of subject matter was noted after the intervention, reflecting a rise in the proportion of papers on vascular, spine, trauma, skull base, and other subjects.

**Table 2:** Type and subject of articles published before and after implementation of residency research program.

Type	Prior (% total)	Post (% total)	P-value
Basic science	4 (22.22)	4 (3.67)	0.0027
Clinical	14 (77.78)	105 (96.33)	0.0027
Subject			
Tumor	5 (27.78)	11 (10.09)	0.03662
Vascular	1 (5.56)	14 (12.84)	0.37346
Spine	0 (0.00)	7 (6.42)	0.267
Trauma	0 (0.00)	3 (2.75)	0.4777
Functional	4 (22.22)	1 (0.93)	< 0.00001
Pediatric	6 (33.33)	34 (31.19)	0.85716
Skull base	2 (11.11)	13 (11.93)	0.92034
Other	0 (0.00)	26 (23.85)	0.2034

Journal characteristics

In total, residents published the greatest number of papers in the Journal of Neurosurgery: Pediatrics, World Neurosurgery, Operative Neurosurgery, and Journal of Neurosurgery (Table 3). Prior to the research program, residents were most frequently published in the Journal of Neurosurgery Pediatrics (n = 3, 16.67%) and the Journal of Neuro-Oncology (n = 3, 16.67%). The Journal of Neurosurgery: Pediatrics (n = 15, 13.76%) and World Neurosurgery (n = 17, 15.60%) were the most frequent afterward.

**Table 3:** Publication count by journal before and after implementation of residency research program.

Journal Title	Number of publications (% total)	Prior (% total)	Post (% total)	P-value
Journal of Neurosurgery: Pediatrics	18 (14.17)	3 (16.67)	15 (13.76)	0.7414
World Neurosurgery	17 (13.39)	0 (0.00)	17 (15.60)	0.3593
Operative Neurosurgery	11 (8.66)	0 (0.00)	11 (10.09)	0.7927
Journal of Neurosurgery	10 (7.87)	1 (5.56)	9 (8.26)	0.34827
Neurosurgery	8 (6.30)	0 (0.00)	8 (7.34)	0.11702
Clinical Neurosurgery	8 (6.30)	0 (0.00)	8 (7.34)	0.11702
Child’s Nervous System	6 (4.72)	1 (5.56)	5 (4.59)	0.42858
Journal of Neurological Surgery, Part B: Skull Base	5 (3.94)	0 (0.00)	5 (4.59)	0.17619
Journal of Neurosurgery: Spine	5 (3.94)	1 (5.56)	4 (3.67)	0.35197
Journal of Neuro-Oncology	3 (2.36)	3 (16.67)	0 (0.00)	< 0.00001
Journal of Neurointerventional Surgery	2 (1.57)	0 (0.00)	2 (1.83)	0.28096
Neuromodulation	2 (1.57)	2 (11.11)	0 (0.00)	0.00022
Neurosurgical Focus	2 (1.57)	1 (5.56)	1 (0.92)	0.07215
American Journal of Medicine	1 (0.79)	1 (5.56)	0 (0.00)	0.00676
Anesthesiology Clinics of North America	1 (0.79)	1 (5.56)	0 (0.00)	0.00676
Current Treatment Options in Oncology	1 (0.79)	1 (5.56)	0 (0.00)	0.00676
Journal of AAPOS	1 (0.79)	1 (5.56)	0 (0.00)	0.00676
Pan Arab Journal of Neurosurgery	1 (0.79)	1 (5.56)	0 (0.00)	0.00676
Seminars in Neurosurgery	1 (0.79)	1 (5.56)	0 (0.00)	0.00676
Academic Radiology	1 (0.79)	0 (0.00)	1 (0.92)	0.3409
American Journal of Neuroradiology	1 (0.79)	0 (0.00)	1 (0.92)	0.3409
BMJ Case Reports	1 (0.79)	0 (0.00)	1 (0.92)	0.3409
Clinical Anatomy	1 (0.79)	0 (0.00)	1 (0.92)	0.3409
Future Cardiology	1 (0.79)	0 (0.00)	1 (0.92)	0.3409
Interdisciplinary Neurosurgery: Advanced Techniques and Case Management	1 (0.79)	0 (0.00)	1 (0.92)	0.3409
Intracranial Aneurysms	1 (0.79)	0 (0.00)	1 (0.92)	0.3409
Journal of Critical Care	1 (0.79)	0 (0.00)	1 (0.92)	0.3409
Journal of Neurological Surgery, Part A: Central European Neurosurgery	1 (0.79)	0 (0.00)	1 (0.92)	0.3409
Journal of Neurosurgical Sciences	1 (0.79)	0 (0.00)	1 (0.92)	0.3409
Journal of Pediatric Neuroradiology	1 (0.79)	0 (0.00)	1 (0.92)	0.3409
Journal of the American College of Surgeons	1 (0.79)	0 (0.00)	1 (0.92)	0.3409
Journal of Trauma and Acute Care Surgery	1 (0.79)	0 (0.00)	1 (0.92)	0.3409
Neurosurgical Review	1 (0.79)	0 (0.00)	1 (0.92)	0.3409
Pediatric Blood and Cancer	1 (0.79)	0 (0.00)	1 (0.92)	0.3409
Pediatric Neurosurgery	1 (0.79)	0 (0.00)	1 (0.92)	0.3409
Pineal Region Lesions: Management Strategies and Controversial Issues	1 (0.79)	0 (0.00)	1 (0.92)	0.3409
PLoS One	1 (0.79)	0 (0.00)	1 (0.92)	0.3409
Radiographics	1 (0.79)	0 (0.00)	1 (0.92)	0.3409
Skull Base Surgery of the Posterior Fossa	1 (0.79)	0 (0.00)	1 (0.92)	0.3409
Spinal Cord Tumors	1 (0.79)	0 (0.00)	1 (0.92)	0.3409
Stroke	1 (0.79)	0 (0.00)	1 (0.92)	0.3409
Surgical Clinics of North America	1 (0.79)	0 (0.00)	1 (0.92)	0.3409
The Chiari Malformations	1 (0.79)	0 (0.00)	1 (0.92)	0.3409

Notably, there was no significant difference in the mean journal impact factor for papers published before and after the research program (Table 4).

**Table 4:** Mean impact factor of journal of publication before and after implementation of residency research program.

	Before	After	P-value
Mean (SD)	3.08 (1.84)	3.01 (1.87)	0.894
Range	0.00–5.93	0.00–10.17	–

## Discussion

The transition from a traditional apprenticeship model of training to a more rounded approach guided by ACGME’s core competencies has created a ripple in the academic culture of neurosurgery [1,3]. This change has resulted in neurosurgical training programs across the world introducing a number of different measures to incorporate research into the curriculum of their residents [19]. While there remains no consensus regarding the most effective way to encourage residents to partake in academic work, medical training programs remain committed to identifying factors that may predict an academic career [7,20-22]. The present study examined this shift in the case of one neurosurgery training program seeking to increase resident scholarly productivity with the development of a research culture.

Our results

Our study represents the first effort to detail the outcomes of an institutional intervention seeking to develop a research culture amongst a group of neurosurgical residents. We found a significant increase in the number of authorships, citation count, and in-residency h-index after the start of this intervention (Figure 1A) with no significant differences in the relevant demographic and pre-residency variables such as gender, PhD status, or number of pre-residency publications to suggest a change in the baseline characteristics of these residents over that period (Table 1).

Furthermore, no significant increase was seen in the proportion of “middle” authorships (Table 1) or mean journal impact factor (Table 4), suggesting that this increase in resident scholarly productivity was achieved without reliance on authorships given without significant contribution or a higher volume of lower impact research. Moreover, by pursuing a wider breadth of clinical research topics, including vascular, spine, trauma, pediatrics, skull base, and others (Table 2), residents were able to publish in a wider breadth of journals than the time prior, including in World Neurosurgery, Neurosurgery, Operative Neurosurgery, and Clinical Neurosurgery, among others (Table 3). This finding likely represents a reflection of the broad subspecialty interests of those involved residents.

Importantly, the development of this research culture was achieved without the enforcement of specific publication targets. These are not associated with increased academic productivity for neurosurgical residents [8]. Nor was there an increase in dedicated research time, departmental resources, or funding, a fact that is relevant to programs seeking to increase the academic productivity of their residents under the constraints of outstanding financial circumstances or clinical obligations. Instead, a research culture was established in this case by emphasizing other established aspects of a supportive educational environment [8]. Our department cultivated a conversation around research by introducing quarterly research meetings into the resident curriculum, in addition to providing medical editing and travel support through the university, thus providing the necessary resources for individuals to achieve their academic goals. In essence, a Research Program Director proved crucial in helping guide residents from a clinical and scientific perspective, the Research Coordinator was invaluable in facilitating adherence to necessary procedures, and the Medical Editor was able to assist with formatting and submission of work. A medical illustrator and statistician were not introduced as residents were encouraged to undertake this work themselves though these additions may have been beneficial.

Prior research

While they have not been associated with increased academic productivity for neurosurgical residents [8], research requirements in general surgery programs have increased in peer-reviewed publications [9,11], accepted abstracts [23], and podium or poster presentations [12]. Interestingly, one program found an increase in presentations but no increase in overall number of publications [12]. While several potential bibliometric measures could be used, we chose to examine peer-reviewed publications to decrease potential misrepresentations of research productivity. For instance, an analysis of applicants for orthopedic surgery found that while applicants report a mean number of 6.7 total publications, only 1.28 of those were peer-reviewed [24]. On the other hand, limiting productivity measures to only the number of peer-reviewed publications may create an erroneous impression of academic impact. Specifically, total publication counts serve as a quantitative measure but fail to consider the variable influence of individual publications [25]. For this reason, we also included h-index and citation counts as additional measures of productivity to quantify the impact of each resident’s research on the field [14,26].

Indeed, meaningful contributions to the field through research provide residents with opportunities for their own professional advancement. Several studies have identified an association between the number of publications during residency and a continued career in academia [6,24]. For neurosurgery residents, Khan et al. found that publishing is one of the most important predictors of promotion in academic medicine [16] and that a supportive research environment is associated with increased publishing [8]. Therefore, educators must begin to consider which factors are likely to contribute most to resident productivity. One program with mandatory research requirements polled residents who considered funding, training, and time to be lacking in pursuit of academic productivity [27,28]. Furthermore, programs with protected research time are more productive across all metrics, including manuscript submissions and publications [8,10]. Miner and Harrington liken this educational obstacle to working on a “fixed income”: if expectations are to be added, what is to be discarded [13]? Going forward, residency programs are anticipated to devise their own solutions to this problem based on the past experiences of other institutions working towards the crucial intersection between systems-based surgical practice and resident research [23].

Limitations

The development of a cultural shift within an organization or institution requires time for full effect after its initial implementation. Therefore, the introduction of the research program has been a process with slow but steady adoption by residents and staff alike, which may not be exactly reproducible by other institutions, thereby limiting its generalizability. This process continues to the present day; however, the recent graduation of a cohort who experienced this program for the entire duration of their residency presents an opportunity to evaluate the impact of this cultural shift.

This is a single-center, single-program study with few variables. While there is no perfect measure of research quality, citation analysis and journal impact factor were used as correlates based on our previous experiences using these metrics to reflect journal readership and interest [7,8,14,16]. Similarly, we acknowledge limitations to data obtained through Scopus and departmental websites, but these methods are also well-established based on our past research. The lack of a reliable protocol to count other scholarly activities beyond the publication of peer-reviewed research may have resulted in underreporting of academic achievement. Furthermore, no post-residency publications were included as they fell outside the scope of this work.

## Conclusions

This study is the first to detail the outcomes of implementing a research culture into a neurosurgery training program. Our results demonstrated an increase in resident academic productivity through structuring a conversation around research within the context of a supportive research environment. As training programs work to meet ACGME requirements for competency in systems-based practice, this experience may serve as a blueprint for future efforts.
